# Genetic profiling of *Mycobacterium tuberculosis* revealed “modern” Beijing strains linked to MDR-TB from Southwestern Colombia

**DOI:** 10.1371/journal.pone.0224908

**Published:** 2020-04-24

**Authors:** Luisa Maria Nieto Ramirez, Beatriz E. Ferro, Gustavo Diaz, Richard M. Anthony, Jessica de Beer, Dick van Soolingen

**Affiliations:** 1 Universidad Santiago de Cali, Cali, Colombia; 2 Departamento de Salud Pública y Medicina Comunitaria, Universidad Icesi, Cali, Colombia; 3 Centro Internacional de Entrenamiento e Investigaciones Médicas (CIDEIM), Cali, Colombia; 4 Universidad Icesi, Cali, Colombia; 5 Mycobacteria Diagnostic Laboratory for Bacteriology and Parasitology (BPD) Center for Infectious Disease Research, Diagnostics and Perinatal Screening (IDS) National Institute for Public Health and the Environment (RIVM), Bilthoven, The Netherlands; 6 Department of Medical Microbiology, University of Groningen, University Medical Center Groningen, Groningen, The Netherlands; 7 Department of Medical Microbiology, Radboud University Medical Center, Nijmegen, The Netherlands; St Petersburg Pasteur Institute, RUSSIAN FEDERATION

## Abstract

Beijing strains of *Mycobacterium tuberculosis* (lineage 2) have been associated with drug-resistance and transmission of tuberculosis worldwide. Most of the Beijing strains identified in the Colombian Pacific coast have exhibited a multidrug resistant (MDR) phenotype. We sought to evaluate the clonality and sublineage of Beijing strains circulating in Southwestern Colombia. Thirty-seven Beijing strains were identified through spoligotyping out of 311 clinical isolates collected in 9 years from 2002–2010. Further analysis by MIRU-VNTR 24 loci was conducted for the Beijing strains. For sublineage classification, deletions of RD105, RD207, and RD131 and point mutations at *fbpB*, *mutT2*, and *acs* were evaluated. Drug-resistance associated mutations to first- and second-line anti-TB drugs were also evaluated. Additionally, two Beijing strains were Illumina-whole genome sequenced (one MDR and one drug-susceptible). Among the 37 Beijing strains characterized, 36 belonged to the SIT190 type from which 28 were MDR, four pre-extensively drug resistant (XDR) TB, and four XDR-TB. The remaining strain was SIT1 and drug susceptible. MIRU-VNTR analysis allowed the identification of three Beijing clusters and two unique strains. Beijing strains were confirmed as “modern” sublineage. The mutations *rpoB* S531L and *katG* S315T were the most common among MDR strains. Moreover, the two strains evaluated by whole genome sequencing (WGS) shared most of the genetic features with the sublineage 2.2.1 “modern” Beijing previously characterized from Asian strains. WGS analysis of the MDR strain revealed the presence of eight SNPs previously reported in other MDR “Beijing-like” strains from Colombia. The presence of “modern” Beijing strains in Southwestern Colombia, most of them with MDR phenotype, suggests a different origin of this *M*. *tuberculosis* sublineage compared to other Beijing strains found in neighboring South American countries. This work may serve as a genetic baseline to study the evolution and spread of *M*. *tuberculosis* Beijing strains in Colombia, which play an important role in the propagation of MDR-TB.

## Introduction

Tuberculosis (TB) is still the leading cause of death worldwide due to a single infectious pathogen, largely because of the dissemination of drug resistant *Mycobacterium tuberculosis (Mtb)* strains [1] The Beijing genotype, one of the most successful and virulent lineages of *Mtb*, has been correlated with active transmission of multidrug resistant (MDR) and extensively drug resistant (XDR) TB [2–5]. Beijing is the major representative strain of the lineage 2 (East-Asian lineage), which is defined by the deletion of region of difference (RD) RD105 [6, 7]. Two different sublineages of Beijing, the “ancient” (atypical) and “modern” (typical) strains have been described. The two variants show differences in geographical distribution, drug resistance, and virulence patterns. The “ancient” sublineage has been found predominantly in Russia, Korea, and Japan [8–10], while “modern” sublineage is distributed worldwide and has been largely associated with drug resistance and hypervirulence [11–13].

Although the prevalence of Beijing strains is not high in South America [14], several countries of the region have now reported the presence of this genotype among clinical isolates [9, 15–18]. In Peru, the proportion of Beijing strains seems to be increasing in the last decade (9% to 16%), with a predominance of the “modern” sublineage, although not highly associated with drug resistance [9, 19]. Similarly, Beijing strains were detected only among pan-drug susceptible TB cases in Paraguay [17]. On the other hand, Beijing strains from Ecuador have been found in both drug susceptible and resistant TB cases, although not clustered [18, 20].

In Colombia, there has been evidence of association between Beijing strains and drug resistance. In 1998, a study conducted in Buenaventura -the main port city on the Colombian Pacific coast- reported a proportion of 10% (11/111) of Beijing strains, three of which were MDR [15]. Later, we identified a cluster of 24 Beijing Spoligo-International-Type (SIT) 190 strains among MDR and XDR *Mtb* isolates in Southwestern Colombia [21, 22]. A high proportion of those Beijing strains were isolated from pulmonary MDR-TB (96%) patients from Buenaventura (92%) [21]. According to the SpolDB4 database, the Beijing SIT190 strains observed in the Colombian Pacific coast have been also observed in the United States, Japan, Cuba, among other countries [23].

Here, we aimed to perform a comprehensive genetic characterization of Beijing strains circulating in Southwestern Colombia, to define its genetic sublineage, clustering and confirm its association with MDR. This work may serve as a genetic baseline to study transmission dynamics and spreading of *Mtb* “modern” Beijing strains in Colombia, which play an important role in the control of MDR-TB.

## Materials and methods

### Ethics statement

The Institutional Review Board of the International Center for Medical Research and Training (CIDEIM) approved this study, authorizing a waiver of informed consent from the human research subjects who provided the samples. Additionally, data was anonymously analyzed, and confidentiality preserved using codes instead of identifiable variables.

### Study samples

**Fig 1:**
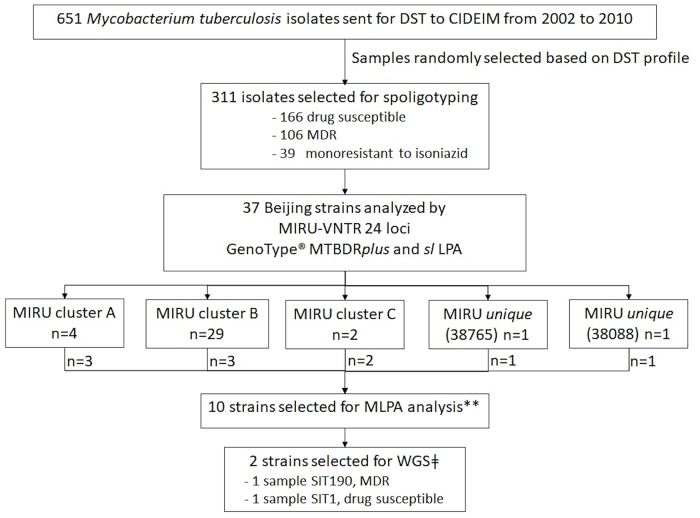
Workflow for the selection of *Mycobacterium tuberculosis* strains to identify and characterize Beijing strains from Southwestern Colombia. DST: Drug susceptibility testing; MDR: Multidrug Resistant; MIRU-VNTR: Mycobacteria Interspersed Repetitive Unit–Variable Number Tandem Repeat; MLPA: Multiplex Ligation-dependent Probe Amplification; LPA: Line probe assays; WGS: Whole Genome Sequencing.

During the period 2002 to 2010, 651 *Mtb* isolates from Valle del Cauca, Colombia were sent to CIDEIM from public and private health institutions. Drug susceptibility testing (DST) was performed by the agar proportion method on Middlebrook 7H10 media for both first (isoniazid, rifampicin, ethambutol, and streptomycin) and second line antibiotics (amikacin, ciprofloxacin or moxifloxacin) [24]. The strains were stored at -80°C. For further characterization, we randomly selected 311 isolates: 106 MDR, 39 monoresistant to isoniazid, and 166 drug-susceptible, to balance the sample set (Fig 1).

### Phylogenetic analysis

All 311 isolates were characterized through spoligotyping as described by Kamerbeek *et al*., [25]. The Beijing genotype was detected in 37 strains, being 25 of them already reported in a previous study [21] and 12 newly identified here. Subsequently, we analyzed the 37 *Mtb* Beijing strains by the high-resolution Mycobacteria Interspersed Repetitive Unit–Variable Number Tandem Repeat (MIRU-VNTR) 24 loci typing [26]. For this purpose, DNA was extracted using the Cetrimonium bromide-CTAB method [27]. Agarose gel electrophoresis was performed to determine the number of repeats for each locus. Finally, we used Quantity one software (Biorad^®^) to determine the length of the PCR products for each of the 24 loci analyzed and for allelic assignation. External quality control was assessed through the participation in the second worldwide proficiency study of MIRU-VNTR [28].

### Beijing strains sublineage identification

In order to confirm the genetic background (Beijing sublineage) and the clonality of these Beijing strains, at least two representative strain of each cluster based on MIRU-VNTR 24 loci classification was selected as well as the two unique types (n = 10) (Fig 1). These analyses included the evaluation of RD105, RD207, RD131 and SNPs at codons *fbpB-238*, *mutT2-58*, and the *acs1551* nucleotide by the Multiplex Ligation-dependent Probe Amplification (MLPA) assay with the readout facilitated by the Luminex bead technology, as previously described [6]. The MLPA analysis was conducted by the Royal Tropical Institute (KIT) in The Netherlands. To classify the Beijing strains into the “modern” or “ancient” sublineages, we followed the algorithm proposed by Bergval *et al*., [6].

### Detection of mutations associated with drug resistance

DNA was extracted according to the manufacturer´s instructions (HainLifescience GmbH, Nehren, Germany). Mutations associated with resistance to first line drugs isoniazid (*katG* and *inhA)* and rifampicin (*rpoB)* were detected using the line probe assay GenoType^®^ MTBDR*plus*. Likewise, mutations associated to second line anti-TB drugs fluoroquinolones (*gyrA*), ethambutol (*embB*), and aminoglycosides (*rrs*), were detected using GenoType^®^ MTBDR*sl* (HainLifescience, Nehren, GmbH, Germany). Mutations were confirmed at KIT, following the SNP detection protocol described by Sengstake *et al*., [29].

### Whole genome sequencing analysis

Based on the drug susceptibility profile, two out of 37 Beijing strains were selected for whole genome sequencing (WGS): one drug-susceptible and one MDR strain. The MDR strain selected for WGS later evolved to XDR. Library preparation and Illumina HiSeq 2000-WGS were performed as previously described [30]. WGS was performed at the Broad Institute. Analysis of Sequence Read Archive (SRA) files was done using the CLC genomics workbench 12 software, having the H37Rv NC_000962.3 as the reference *Mtb* sequence.

### Data analysis

The SIT number and the 24 Multi Locus Variable-number-tandem-repeat Analysis (MLVA) were determined using the http://www.miru-vntrplus.org web page and SITVIT2 (database of the Pasteur Institute of Guadeloupe) [31]. The information obtained was used to build the phylogenetic analysis. VNTR typing results were compared to the database at the National Institute for Public Health and the Environment (RIVM) in the Netherlands.

## Results

### Samples characteristics

From 2002 to 2010, we identified 37 Beijing strains from *Mtb* isolates collected in Southwestern Colombia by spoligotyping (Fig 1). Phenotypic DST showed that 30 of these strains were MDR, six XDR, and only one was drug susceptible to all drugs evaluated. Beijing strains were found more frequently among female (23/37, 62%) and young patient´s, with a median age of 29 years (IQR: 24–40 years). Most of the Beijing isolates were from pulmonary TB cases (34/35, 97.1%); 39% (14/36) were new cases exhibiting MDR (n = 12) or XDR (n = 2) phenotype. Likewise, most of the Beijing strains were isolated from patients from Buenaventura (86%, 32/37) (Table 1 and S1 Table).

**Table 1:** Characteristics of TB patients and 24-loci MIRU-VNTR Beijing clusters.

Characteristics	Total of Beijing strains (n = 37)	Beijing classification by MIRU				
		Cluster A (n = 4)	Cluster B (n = 29)	Cluster C (n = 2)	Unique (38765)	Unique (38088)
**MLPA**^a^ **analysis**	10	3	3	2	1	1
**Sex**						
Female	23 (62.2%)	2	20	0	0	1
Male	14 (37.8%)	2	9	2	1	0
**Origen**						
Buenaventura	32 (86.5%)	4	24	2	1	1
Other	3 (8.1%)	0	3	0	0	0
NA	2 (5.4%)	0	2	0	0	0
**Age (years)**						
0–16	4 (10.8%)	0	4	0	0	0
17–31	16 (43.2%)	0	15	0	1	0
32–50	11 (29.8%)	2	7	1	0	1
>50	4 (10.8%)	1	2	1	0	0
NA	2 (5.4%)	1	1	0	0	0
**Clinical presentation**						
Pulmonary	34 (91.9%)	4	26	2	1	1
Extrapulmonary	1 (2.7%)	0	1	0	0	0
NA	2 (5.4%)	0	2	0	0	0
**Condition**						
New	15 (40.6%)	3	10	1	0	1
Previously treated	13 (35.1%)	1	11	0	1	0
NA	9 (24.3%)	0	8	1	0	0
**Drug susceptibility profile**						
MDR	32 (86.5%)	4	26^b^	1	1^c^	0
XDR	4 (10.8%)	0	3	1	0	0
Drug Susceptible	1 (2.7%)	0	0	0	0	1

NA: Information not available. MIRU-VNTR: Mycobacteria Interspersed Repetitive Unit–Variable Number Tandem Repeat.

^a^. Number of strains selected for Multiplex Ligation-dependent Probe Amplification (MLPA).

^b^. Two patients later evolved to XDR-TB.

^c^. This patient evolved to XDR-TB.

### Phylogenetic classification

**Fig 2:**
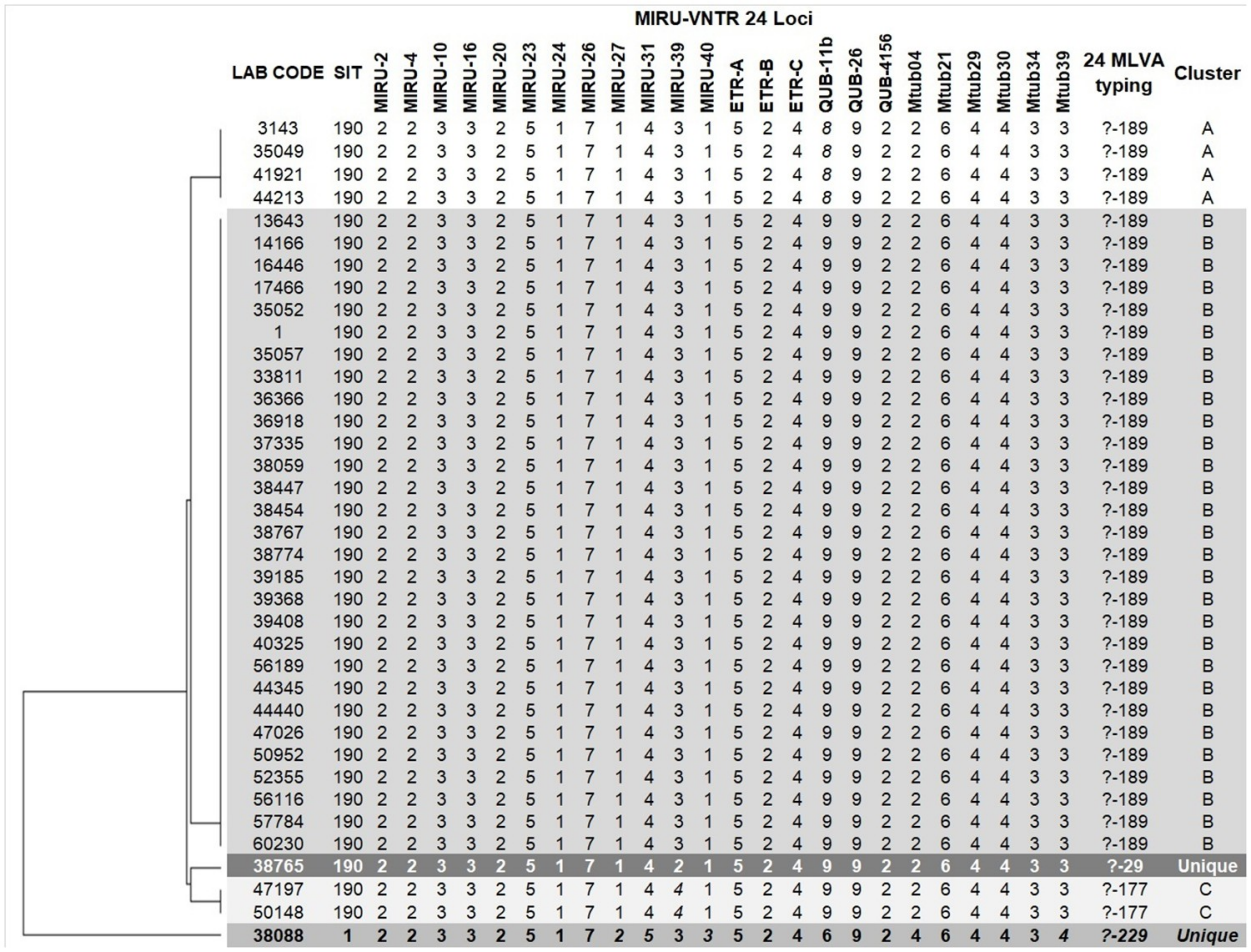
Unweighted pair group method with arithmetic mean (UPGMA) tree of the Beijing strains discriminated by spoligotyping and MIRU-VNTR. 24 Multi locus variable-number-tandem-repeat analysis (MLVA) typing refers to the 15 most discriminatory and 9 ancillary loci defined by Weniger *et al*., 2010 (31). Interrogation mark represent genotypes not previously reported or orphan. Clusters A to C defined based on MIRU analysis. MIRU-VNTR: Mycobacteria Interspersed Repetitive Unit–Variable Number Tandem Repeat.

All except one Beijing strain were typed as SIT190 (000000000003731), the remaining drug-susceptible strain was typed as SIT1 (000000000003771). MIRU-VNTR 24 loci analysis discriminated the 37 Beijing strains into three clusters, A to C, and two unique strains (Table 1), with a clustering rate of 86.5%, calculated with the formula presented by Meehan *et al*, 2018 [32]. Overall, two loci (QUB11b and MIRU39) exhibited the highest allelic diversity in the SIT190 Beijing strains (Fig 2). Specifically, MIRU39 revealed the highest differences, with two, three, and four repeats among the Beijing SIT190 strains. In addition, five more loci (Mtub39, Mtub04, MIRU31, 27, and 40) showed different number of repeats between SIT190 and SIT1 (Fig 2). The discriminatory power index of MIRU-VNTR 24 loci (*D* = 0.3799) was superior compared to the discriminatory power index of spoligotyping (*D* = 0.0541) as defined by Hunter and Gaston [33].

### Beijing strains sublineage

Based on the phylogenetic analysis derived from MIRU-VNTR 24-loci analysis, we selected 10 representative samples of each cluster, as follows: cluster A = 3, B = 3, C = 2, and the two unique strains (Figs 1 and 2). The East/Asian RD105 deletion was present in all ten Beijing strains, confirming their identification as members of the Beijing lineage 2 (or East Asian lineage). These representative set of samples was further classified as “modern” Beijing sublineage clade Vietnam (V+)/China (CHIN+) based on intact RD207 and RD131, together with the SNPs at *fbpB238* and *mutT2-58* (Table 2) [6, 7].

**Table 2:** Results of molecular markers for Beijing classification.

Strain number	Molecular markers for Beijing classification						
	RD105 deletion	*fbpB* 238	RD207 deletion	*mutT2 58*	*acs 1551(G/A)*	RD131 deletion	Beijing classification^a^
**35049**	Positive	CCC to CCA^b^	Negative	GGA to CGA^c^	Negative	Negative	Clade V+, CHIN+
**41921**	Positive	CCC to CCA^b^	Negative	GGA to CGA^c^	Negative	Negative	Clade V+, CHIN+
**44213**	Positive	CCC to CCA^b^	Negative	GGA to CGA^c^	Negative	Negative	Clade V+, CHIN+
**47026**	Positive	CCC to CCA^b^	Negative	GGA to CGA^c^	Negative	Negative	Clade V+, CHIN+
**36366**	Positive	CCC to CCA^b^	Negative	GGA to CGA^c^	Negative	Negative	Clade V+, CHIN+
**36918**	Positive	CCC to CCA^b^	Negative	GGA to CGA^c^	Negative	Negative	Clade V+, CHIN+
**50148**	Positive	CCC to CCA^b^	Negative	GGA to CGA^c^	Negative	Negative	Clade V+, CHIN+
**47197**	Positive	CCC to CCA^b^	Negative	GGA to CGA^c^	Negative	Negative	Clade V+, CHIN+
**38765**	Positive	CCC to CCA^b^	Negative	GGA to CGA^c^	Negative	Negative	Clade V+, CHIN+
**38088**	Positive	CCC to CCA^b^	Negative	GGA to CGA^c^	Negative	Negative	Clade V+, CHIN+

CHIN: China, V: Vietnam.

^a^. According to Bergval algorithm.

^b^. Synonymous mutation.

^c^. Gly58Arg.

### Mutations associated with drug resistance

A total of 33 (29 MDR and 4 XDR) out of the 37 Beijing strains were successfully evaluated for the presence of mutations associated with first- and second-line drug resistance (Table 3 and S1 Table). Three Beijing *Mtb* strains were excluded from the analysis because they did not hybridize the amplification control band for either first- or second-line probe assay. Additionally, three strains did not hybridize the amplification control band for GenoType MDR*sl* only, while two additional strains did not have a valid result for the *rrs* gene. The susceptible strain was not tested. The mutations S531L in *rpoB* and S315T in *katG*, associated with resistance to rifampicin and isoniazid, respectively, were the most frequently found in MDR cases (Table 3).

**Table 3:** Frequency of mutations associated with drug resistance in “modern” Beijing strains from Southwestern Colombia.

Gene mutation probe (mutation)	% of strains (# genotypically resistant /# tested strains by LPA)	Drug
***rpoB* MUT3 (S531L/tcg>ttg)**	97 (32/33)	Rifampicin
***katG* MUT1 (S315T/agc>acc)**	100 (33/33)	Isoniazid
***inhA* MUT1 (c-15t**^a^**)**	3 (1/33)	Isoniazid/Ethionamide/Prothionamide
***gyrA* MUT3C (D94G/ gac>ggc)**	13 (4/30)^b^	Fluoroquinolones
***rrs* MUT1 (a1401g)**	29 (8/28)	Aminoglycosides
***embB* MUT1B (M306V/atg>gtg)**	91 (29/30)	Ethambutol

DST: drug susceptibility testing by the agar proportion method. LPA: Line Probe Assay. Lower case indicates the nucleotide changes while uppercase letter indicates aminoacid change.

^a^. Mutation in the regulatory region.

^b^. One strain had double mutations MUT3A (D94A) and MUT3C (D94G).

All four XDR strains were confirmed using the genotype analysis that also allowed the detection of four additional pre-XDR strains (defined as an MDR strain that is also resistant to either a fluoroquinolone or a second-line injectable) [34]. The SNP M306V in the *embB* gene was the most frequently encountered among the ethambutol resistant strains (Table 3).

### Whole genome sequencing analysis

Two Beijing strains were analyzed in duplicate by WGS, 38088 (drug susceptible) and 38765 (MDR). The genome data are available at the National Center for Biotechnology Information (NCBI) under the BioProject identifiers PRJNA227751 and PRJNA227755 respectively. SRA data SRS565195 and SRS565201 were used for the SNP detection analysis. The average coverage was 23X for the MDR Beijing (38765) and 55X for the drug susceptible Beijing (38088).

WGS analysis confirmed the SNPs at *mutT2-58* and *fbpB-238* genes for the “modern” Beijing sublineage classification (Table 2). We also found a total of 44 and 72 SNPs previously reported in the “modern” and 2.2.1 sublineage Beijing previously reported in Asian countries (S2 Table). These mutations include the C1477596T SNP (*ogt* at codon 12).

All SNPs associated with the MDR phenotype were confirmed for the strain 38765. WGS of the MDR strain also allowed the identification of eight SNPs that had been previously reported in MDR Beijing strains from Colombia by Rodriguez-Castillo *et al*., [35] (Table 4). Among other relevant SNPs, both Beijing strains (38088 and 38765) exhibited an insertion of a Guanine at the position 1406760^1406761 in the *Rv1258c* gene. There was also an exclusive mutation found in the strain 38088, the synonymous SNP in the *cysA3* gene.

**Table 4:** Comparison of the previously reported SNPs in Beijing-like strains from Colombia and the Beijing strains of this study.

Accession number	Gene name	Position	Reference H37Rv	Mutation in other Beijing strains from Colombia (35)	Mutation in MDR Beijing strain 38765	Mutation in DS Beijing strain 38088
**Rv0355c**	*PPE8*	434226	A	Deletion	Wild type	Wild type
**Rv0355c**	*PPE8*	434227	C	Wild type	Deletion	Wild type
**Rv0197**	*Rv0197*	233949	C	T	T	Wild type
**Rv0753c**	*mmsA*	845542	G	A	A	Wild type
**Rv0988**	*Rv0988*	1106176	A	C	C	Wild type
**Rv1723**	*Rv1723*	1950068	C	T	T	Wild type
**Rv2308**	*Rv2308*	2580877	G	T	T	T
**Rv2940c**	*Rv2940c*	3279637	G	A	A	Wild type
**Rv3806c**	*ubiA*	4269304	A	C	C	Wild type
**Rv3862c**	*whiB6*	4338365	A	G/C	G	Wild type

DS: Drug susceptible. MDR: Multidrug Resistant

## Discussion

Modern Beijing sublineage has been associated with emergence and dissemination of drug resistance TB and higher virulence worldwide (10). Here, we reported the identification of 37 *Mtb* “modern” Beijing strains isolated from Southwestern Colombia TB cases in a 9-year period. Most of these “modern” Beijing strains (36/37) were MDR or XDR, strongly suggesting an association with drug resistance. Interestingly, a large proportion of the drug resistant strains were isolated from TB patients from Buenaventura (S1 Table and Table 1), confirming previous findings that suggested Buenaventura as a hotspot for MDR-TB and the *Mtb* Beijing linage [21, 22]. The highly homogeneous Beijing-MDR cluster SIT190 was further discriminated in three clusters by MIRU-VNTR 24-loci typing (Fig 2). The homogeneity observed in the “modern” Beijing strains from Southwestern Colombia, and the fact that most of them were isolated from new (not-previously treated) TB patients (40.6%, Table 1), suggest active transmission of these MDR strains.

Beijing strains (mostly, SIT190) isolated in Colombia have shown highly drug-resistant phenotypes and this varies from the Beijing strains isolated in other South American countries, where there is not frequent association with drug resistance thus far [9, 14, 19]. Our findings support the hypothesis that different Beijing strains were introduced at different occasions in South American countries, most likely reflecting the diversity in human migration since ancient times [12]. This is also in agreement with the findings of Schürch, *et al*., on the introduction of multiple sources of spread of Beijing strains to different geographical areas on many different occasions [36]. The “modern” Beijing strains isolated in Colombia are highly conserved and this may reflect their recent introduction and spreading.

Recently, Beijing genotype was found in *Mtb* strains from two indigenous patients from a community in the Colombian pacific region. These Beijing strains belonged to SIT190 and SIT406, one of them being MDR [37]. Additionally, from 2013 to 2015, two Beijing strains were found in Cali, the most populated city in the Colombian pacific area [38]. Collectively, these findings suggest the potential spreading of Beijing strains from Buenaventura to other cities in the Colombian pacific region, with the likelihood to cause MDR-TB cases, and the need to strengthen surveillance strategies.

We detected a high frequency of young female TB patients infected with Beijing strains. This distribution was particularly observed in the cluster “B” identified by MIRU-VNTR typing (Table 1). This finding agrees with previous reports from Malawi, where there were more women than men infected with Beijing strains, albeit without association with drug resistance [4, 39]. Of note, the “modern” Beijing sublineage was associated with female young patients and drug resistance in a recent study from Vietnam [40], as we observed in our study. Additionally, an important trend between female and MDR-TB in patients from South America and other geographical areas has been reported [41–44]. The link we found between female and MDR “modern” Beijing strains is intriguing since *Mtb* has affected mostly men specially in low and middle-income countries [45] and there is also evidence of some Beijing strains affecting more frequently men than women (2:1 ratio approximately) [4, 46, 47].

WGS analyses of two isolates (One SIT190/MDR and one SIT1/drug susceptible) confirmed the “modern” Beijing sublineage. Although, we only have the data for two out of the 37 Beijing strains, both strains shared all the 72 and 44 SNPs with the 2.2.1 “modern” sublineage of Beijing (S2 Table) mostly identified from China, Russia and Thailand [10, 48]. These additional genetic descriptions revealed great similarity of the Beijing strains isolated in Colombia with “modern” Beijing strains from Asia instead of the Pacific or Asian-African Beijing sublineages [48]. Additionally, our MDR strain that was sequenced shared the eight SNPs that were proposed to be exclusively present in Beijing MDR strains from Colombia (Table 4) [35]. On the other hand, the drug-susceptible Beijing strain only shared one mutation with another “Beijing-like” strains from Colombia (Table 3) [38]. This SNP was in the *Rv2308* gene that encodes a conserved hypothetical protein that may act as a transcriptional regulator [49]. Importantly, the SNP found in *Rv2308* has not been identified in other Beijing strains from South Africa or Asia [36].

A large proportion of our Beijing strains were phenotypically resistant to streptomycin (33/37 strains) (S1 Table). WGS showed a SNP at *Rv1258c* that encodes for an efflux pump, probably linked to streptomycin resistance. This SNP was previously identified in “modern” Beijing strains from Guatemala [50].

We acknowledge several limitations of the present study. The study design included a convenience sampling with a limited number of Beijing isolates that were available in a repository from Colombia. Nevertheless, the samples included all Beijing strains found in this setting during a 9-years period. Additionally, the repository was built over the years based on cultures sent to CIDEIM for surveillance purposes. Due to budget constraints, only a few samples were analyzed by all the methodologies, yet the study strategy achieved the representativeness sought. Although there is not consensus on which SNPs are most informative and reliable to accurately describe the phylogeny of Beijing genotype [7], we could successfully classify our Beijing strains into the “modern” sublineage; following the algorithm proposed by Bergval *et al*., [6]. Information obtained by high throughput technologies, like WGS, will shortly improve our understanding and definition of Beijing strains circulating locally and worldwide, but such technologies are not readily available in all settings.

In conclusion, Beijing strains that circulated in Colombia from 2002 to 2010 belong to a genetically conserved cluster of “modern” Beijing strains, and have important phenotypic differences with Beijing strains circulating in other South American countries (most of them being MDR strains). Surveillance and monitoring of the local *Mtb* population structure are key strategies to facilitate the development of specific measures to control MDR-TB.

## Supporting information

S1 Table Phenotypic drug susceptibility profile and epidemiological data of the Beijing TB cases from Colombia. (XLSX)

S2 Table SNPs found in Beijing strains from Colombia that were shared with previous WGS studies of Beijing strains worldwide. (XLSX)
